# Sunflower: a potential fructan-bearing crop?

**DOI:** 10.3389/fpls.2015.00798

**Published:** 2015-10-12

**Authors:** Giselle M. A. Martínez-Noël, Guillermo A. A. Dosio, Andrea F. Puebla, Ester M. Insani, Jorge A. Tognetti

**Affiliations:** ^1^Instituto de Investigaciones en Biodiversidad y Biotecnología-Consejo Nacional de Investigaciones Científicas y TécnicasMar del Plata, Argentina; ^2^Laboratorio de Fisiología Vegetal, Facultad de Ciencias Agrarias, Universidad Nacional de Mar del PlataBalcarce, Argentina; ^3^Instituto de Biotecnología, CICVyA-CNIA-INTAHurlingham, Argentina; ^4^Comisión de Investigaciones Científicas de la Provincia de Buenos AiresLa Plata, Argentina

**Keywords:** sunflower, fructan regulation, 1-SST, 1-FFT, sucrose

## Abstract

Grain filling in sunflower (*Helianthus annuus* L.) mainly depends on actual photosynthesis, being the contribution of stored reserves in stems (sucrose, hexoses, and starch) rather low. Drought periods during grain filling often reduce yield. Increasing the capacity of stem to store reserves could help to increase grain filling and yield stability in dry years. Fructans improve water uptake in soils at low water potential, and allow the storage of large amount of assimilates *per* unit tissue volume that can be readily remobilized to grains. Sunflower is a close relative to Jerusalem artichoke (*H. tuberosus* L.), which accumulates large amounts of fructan (inulin) in tubers and true stems. The reason why sunflower does not accumulate fructans is obscure. Through a bioinformatics analysis of a sunflower transcriptome database, we found sequences that are homologous to dicotyledon and monocotyledon fructan synthesis genes. A HPLC analysis of stem sugar composition revealed the presence of low amounts of 1-kestose, while a drastic enhancement of endogenous sucrose levels by capitulum removal did not promote 1-kestose accumulation. This suggests that the regulation of fructan synthesis in this species may differ from the currently best known model, mainly derived from research on Poaceae, where sucrose acts as both a signaling molecule and substrate, in the induction of fructan synthesis. Thus, sunflower might potentially constitute a fructan-bearing species, which could result in an improvement of its performance as a grain crop. However, a large effort is needed to elucidate how this up to now unsuspected potential could be effectively expressed.

## Introduction

Sunflower (*Helianthus annuus* L.) is an important crop worldwide, ranking 4^th^ among oil producing species^1^ which produces high quality oil for human consumption. In this species, grain filling mainly depends on actual photosynthesis. According to a detailed C budget analysis (Hall et al., 1990), C fixed during the pre-anthesis period contributed just about 15% of the total C uptake of the grain in plots without water deficiencies. Increasing the capacity of stem tissues to store reserves could help to increase grain filling and specially yield stability. In general, grains located at the inner part of the capitulum (this is, the youngest ones) fail to develop properly, leaving the center of the harvested organ with a majority of vain grains. This phenomenon is known to depend on photoassimilate availability (Alkio et al., 2003; Cantagallo et al., 2004) since deficient vascular connections do not prevent seed filling in sunflower (Alkio and Grimm, 2003). In fact, the situation is frequently aggravated by the occurrence of drought periods during grain filling, which are typical of one of the main sunflower regions in the world such as Argentinean Pampas (Sadras and Hall, 1989). Under these conditions, photosynthetic rate of sunflower plants may be severely diminished. Hall et al. (1989, 1990) reported that C fixed during the pre-anthesis period contributed about 27% of the total C uptake of the grain in water stressed crops. In spite of this greater C remobilization, during a water stress period this may not be enough to compensate for impaired C fixation since physiological processes such as photosynthesis and growth are affected as well. Thus, severe yield losses are often encountered in dry years (Sadras et al., 1993). A decrease in grain quality is also expected as both the relative oil content and fatty acid composition depend on intercepted PAR (photosynthetically active radiation) during grain filling (Dosio et al., 2000; Izquierdo et al., 2009).

Stored sugars in sunflower stem consist of sucrose and mono-saccharides, which together with a limited amount of starch constitute the reserves that could be remobilized to grains (Goldschmidt and Huber, 1992; Sims et al., 1999). Since C storage in the form of mono and disaccharides implies a high increase in cell osmolality, accumulation of these sugars in stems leads to a rapid increase in cell and tissue volume, which is revealed mainly by stem thickening. Thus, part of C surplus is diverted to investment in structural components, instead of remaining as reserve carbohydrates which could be remobilized to the capitulum. The polymerization of sucrose into fructans could give the possibility of storing much larger amount of assimilates *per* unit issue volume without a significant osmotic effect. Accordingly, by introducing fructosyltransferase genes into sugarcane plants, Nell (2007) found sugar content to be up to 63% higher than in control, untransformed plants. Stored fructans are in turn readily mobilized when necessary. In most small grain crops, fructans are stored in the stem and then contribute to grain filling in a higher degree than sucrose does in sunflower. For example, Borrell et al. (1989) reported that post-anthesis stem reserves may have contributed at least 21% of final grain yield of semi-dwarf wheat, while Gebbing et al. (1999) reported similar average values for two cultivars of this species. These percentages are increased during periods of drought (Blum, 1998; Gupta et al., 2011). Because reserve assimilates stored in vegetative organs of the plant before anthesis may be mobilized during grain filling, they may buffer grain yield against adverse conditions for photosynthesis during that period. This is a general phenomenon observed in cereals (Gebbing et al., 1999), and other crops including sunflower (Hall et al., 1989). Besides its putative effect on C storage and remobilization to reproductive structures, the possibility that sunflower accumulates fructan could also have a direct impact on crop tolerance to summer drought. It has been demonstrated that sunflower capacity for osmoregulation is closely related to yield maintenance in dry years (Chimenti et al., 2002). Fructans are known to provide osmotic regulation to plants, since they are present as a continuum of oligomers that differ from each other in one fructose residue, and they can be easily depolymerized into hexoses. This feature enables fructan-bearing plants to extract water at lower soil water potentials (Garcia et al., 2011). Moreover, fructans are known to protect membranes by direct insertion within lipids and thus relief plants from drought stresses (Valluru and Van den Ende, 2008; Livingston et al., 2009; Van den Ende, 2013).

During the last two decades several attempts have been made to transfer fructan genes from fructan-bearing species to other plants. As a result, improved drought or freeze resistance have been reported in several cases (Pilon-Smits et al., 1995; Konstantinova et al., 2002; Hisano et al., 2004; Parvanova et al., 2004; Bie et al., 2012) but as a general rule the success of such transformations has been rather limited since transgenic plants tend to produce fructan in a low concentration (Cairns, 2003). Furthermore, in sunflower this possibility is uncertain since this species has been considered as recalcitrant for genetic transformation, because of difficulties in plant regeneration procedures (Moschen et al., 2014).

Sunflower is a close relative to Jerusalem artichoke (*H. tuberosus* L.), which accumulates large amounts of fructan of the inulin type in tubers (which are modified stems) but also in true stems and even in roots (Seiler, 2007). In fact, Jerusalem artichoke (as a sugar crop) can be grown for both tubers and aboveground biomass, for which there are cultivars characterized by well-developed stalks and low tuber yields (Caserta and Cervigni, 1991). The reason why sunflower does not accumulate fructans is obscure. Early in the 20^th^ century M. H. Colin in France performed grafting experiments between sunflower and Jerusalem artichoke which revealed that only the parts belonging to the latter accumulated inulin, irrespective of which species was at the base of the graft (Colin, 1922). A first possibility to explain the lack of fructan in sunflower is that genes for fructan synthesis enzymes may be missing, or non-functional, in this species. A second possibility is that, even if present, expression of fructan synthesizing enzymes in sunflower is inhibited because of metabolic reasons. Both possibilities are analyzed below.

## Presence of Fructan Synthesizing Enzymes in Sunflower

An exhaustive analysis regarding the possibility that genes for fructan synthesis enzymes in sunflower may be lacking, or non-functional, has not been conducted yet. This may be attributed to the fact that the sunflower genome is still not available. The accepted model for fructan synthesis in higher plants involves the presence of distinct enzymes, mainly including 1-SST (1-sucrose:sucrose fructosyltransferase) and 1-FFT (fructan:fructan 1-fructosyltransferase) for fructan initiation and polymerization respectively, (Vijn and Smeekens, 1999). With the goal of investigating whether sunflower genome encodes fructan synthesizing enzymes we searched for homologous sequences of 1-SST and 1-FFT from different plant species within a public sunflower EST database^2^ (Bioinformatics Unit at INTA, Hurlingham, Buenos Aires, Argentina). From several contigs identified that showed high similarity with invertases (INV) and fructosyltransferases from several plant species genes, two showed high similarity to fructan synthesis enzyme genes: HeAn_C_8450_Contig8450 (Hacontig8450) to 1-SST and HeAn_C_12894_Contig12894 (Ha12894) to 1-FFT (**Table 1**). It was previously described that development of transfructosylation ability in INV is evidenced by different amino acid substitutions, e.g., W to Y or F and/or N to S in the WMNDPNG motif, and WGW to WGY, or WGF (Lammens et al., 2012). While most of the contigs we found seem to belong to the INV family (e.g., HeAn_C_12706_Contig12706, Ha12706), Hacontig8450 and Ha12894 lack all the essential amino acids that characterize INV enzymes (**Table 1**) suggesting that they belong to fructosyltransferases group.

**Table 1:** Multiple alignment of amino acids of a selection of plant 1-sucrose:sucrose fructosyltransferase (1-SST), fructan:fructan 1-fructosyltransferase (1-FFT), and invertases (INV) enzymes showing essential motifs in the vicinity of the active site.

*Helianthus tuberosus* 1-SST (AJ009757)	FISDPDG	WGN	MTGSAT	QVQ	DEDR	WGY	GWAN
*Cichorium intybus* 1-SST (JQ346799)	FISDPDG	WGN	MTGSAT	QLQ	DEDR	WGY	GWAN
Hacontig8450	FISDPDG	WGN	MTGSAT	QVQ	DEDR	WGY	GWAN
*Cynara scolymus* 1-SST (Y09662.1)	YISDPDG	WGN	MTGSAT	QLQ	DEDR	WGY	GWAN
*Allium cepa* 1-SST (AJ006066)	FMADPNA	WDY	WSGYAT	QVQ	DDER	WGY	GWAS
*Triticum aestivum* 1-SST (AB029888)	YQNDPNG	WEP	LTGSIT	QVT	DDDR	WAY	GWAN
*Lolium perenne* 1-SST (AA086693)	YMNDPNG	WGN	LTGSIT	QVQ	DDER	WAY	GWAN

*H. tuberosus* 1-FFT (AJ009756)	FIYDPDG	WGN	LSGSTT	QLQ	EGHG	WGY	GWAT
*C. intybus* 1-FFT (U84398)	FIYDPNG	WGN	LSGSTT	QLQ	EGHG	WGY	GWAT
*C. scolymus* 1-FFT (AJ000481)	FIYDPNG	WGN	LSGSTT	QLQ	EGHA	WGY	GWAT
**Hacontigl2894**	**FIYDPNG**	**WGN**	**LSGSTT**	**QLQ**	**EGHG**	**WGY**	**GWAT**
*T. aestivum* 1-FFT-A (AB088409)	YQNDPNG	WGN	LTGSIT	QVT	DDDR	WGY	GWAN
*T. aestivum* 1-FFT-B (AB088410)	YQNDPNG	WGN	LTGSIT	QVT	DDDR	WGY	GWAN

*Solanum tuberosum* INV (AEV46297)	WMN NDPNG	WGN	WTGSAT	QVQ	DDNK	WGW	GWAS
*Agave tequiliana* INV (AFJ21575)	WMN NDPNG	WGN	WSGSAT	QVQ	SDDN	WGW	GWAS
**Hacontigl2706**	**WMN NDPNG**	**WGK**	**WTGSAT**	**QVQ**	**DDDR**	**WSW**	**GWAS**

*Underlined letters indicate amino acid substitutions that confer transfructosylation ability. Gray boxes show amino acids that are essential for vacuolar INV activity, which are not present in fructan synthesizing enzymes. Contigs from *H. annuus* are shown in bold*.

## Regulation of Fructan Synthesizing Enzymes in *Helianthus*

The best known model for the induction of fructan synthesis has been developed from research on Poaceae such as wheat and barley. According to the model, sucrose must exceed a certain threshold to elicit the expression of fructan synthesizing enzymes (Pollock et al., 2003). Therefore, sucrose appears to play a double role in fructan metabolism, it is both the essential substrate used in fructan synthesis and it also starts the signal transduction pathway that induces the fructosyl-sucrose synthesizing enzymes (Tognetti et al., 2013). It could be possible that, in the case of sunflower, insufficient sucrose levels are accumulated for 1-SST induction, either as a consequence of a higher threshold for sucrose-driven induction in sunflower than in Jerusalem artichoke, or to a high fructan hydrolase activity which could preclude fructan from accumulating in sunflower tissues.

**FIGURE 1:**
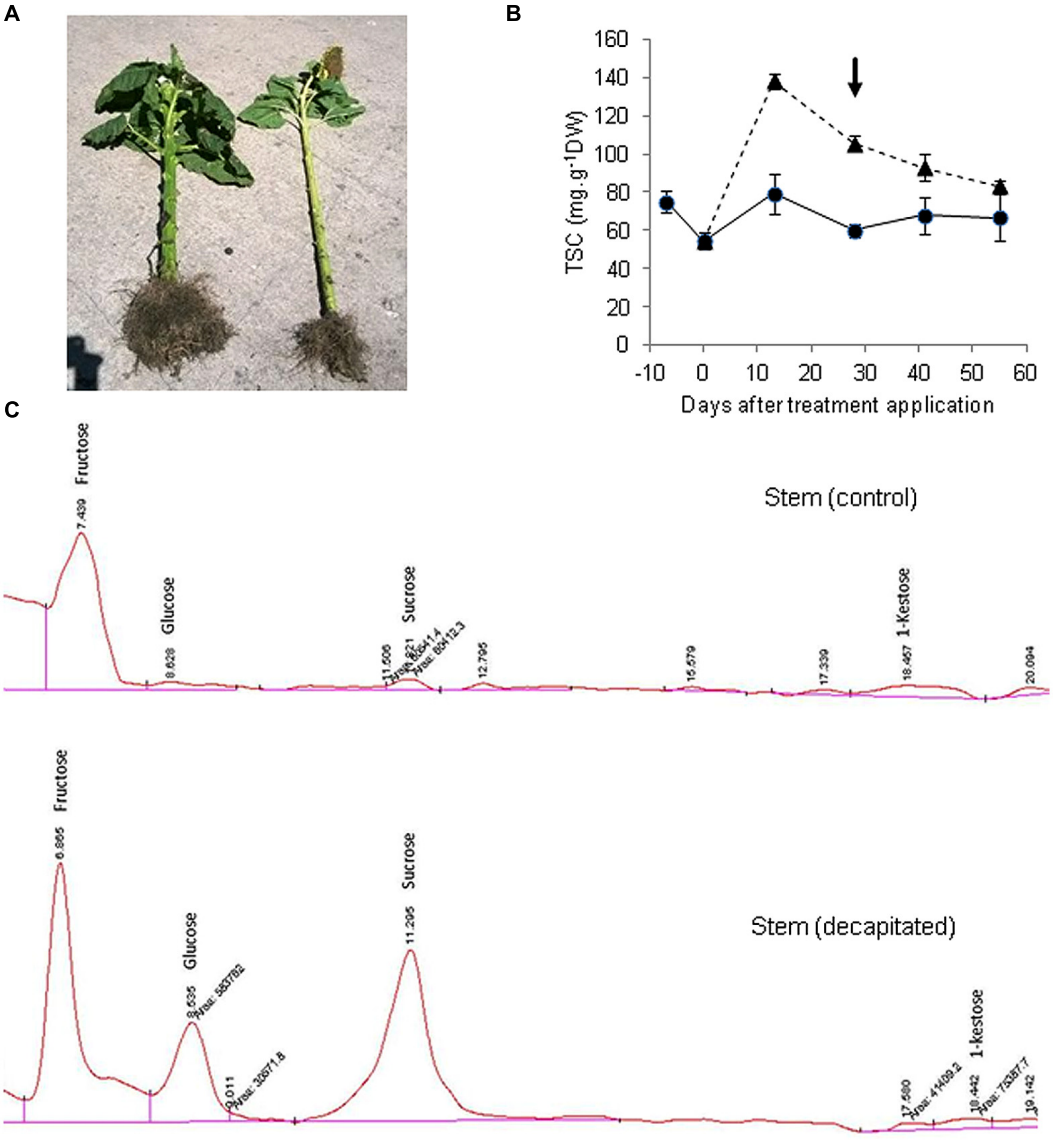
**Sugar accumulation and profiles in sunflower stems from control and decapitated plants. (A)** View of a decapitated plant (left) showing large stem thickening and generalized growth promotion, in comparison to a control plant (right). **(B)** Total soluble carbohydrate concentration (TSC) in stems of decapitated (triangles, dashed line) and control (circles, full line) plants (±SE), measured spectrophotometrically from aqueous extracts, according to the phenol-sulfuric method (arrow indicates sampling date). **(C)** HPLC chromatograms of aqueous stem extracts separated on a Prevail carbohydrates ES column and detected by refractive index detector (RID).

We subsequently performed an experiment in which endogenous sucrose levels in sunflower stems were drastically enhanced by removing the main sink organ (capitulum), to analyze a possible induction of fructan synthesis. A commercial sunflower hybrid (VDH481, Advanta Semillas SAIC) was grown in the field at Balcarce, Argentina, under potential conditions for this environment. When the crop had reached Schneiter and Miller’s R3 phenological stage (about 120° days before anthesis, considering a base temperature of 6°C) half of the plants were decapitated. This treatment resulted in both a large (152%) increase in stem diameter, and enhanced (>24-fold) total sugar concentration in stem tissues, relative to intact plants (**Figures 1A,B**). Stem sucrose concentration of decapitated plants ranged between 5.73 and 7.45 mg sucrose g^-1^ FW, depending on sampling date. Thus, it may be hypothesized that the large tissue expansion driven by decapitation precluded sucrose from reaching concentrations that could have exceeded 15 mg g^-1^ FW if stem volume had remained unchanged. Sucrose threshold values for fructan induction in grass species close to 15 mg g^-1^ FW have been reported (Koroleva et al., 1998), although a straightforward comparison between species is not feasible due to stem volume change in sunflower. A HPLC analysis of sugar composition revealed the presence of 1-kestose in stem of control plants, but its levels were not increased by capitulum decapitation despite the large accumulation of sucrose (**Figure 1C**), reaching 0.24 mg g^-1^ FW. On the other hand, non-significant amounts of oligomers with a higher degree of polymerization were found (not shown).

The inability of sunflower to substantially accumulate fructan is likely not atributtable to a high fructan hydrolase activity, since the latter enzyme is known to be inhibited by sucrose in most species (Van Riet et al., 2006). On the other hand, it is possible that in sunflower (and perhaps in Asteraceae in general) sucrose alone is not sufficient to induce synthesis of fructans, in contrast to monocots. In this sense, working with chicory hairy root cultures, Kusch et al. (2009) showed that in addition to high levels of sucrose, low N levels are required to induce fructan synthesizing enzymes. Furthermore, from work on *H. tuberosus* it appears as possible that fructan synthesis initiation may be related to the induction of tuberization, because no polymerization of sucrose appears to occur before the beginning of tuber growth (Monti et al., 2005). The onset of tubering is hormonally regulated, being jasmonic acid a well-known promoter (Koda et al., 1994). Deepening our knowledge on *H. tuberosus* biology with studies such as that by Jung et al. (2014) may also help find the responses.

## Conclusion and Future Prospects

Our results suggest that the inability of sunflower to substantially accumulate fructan is not attributable to the lack of functional genes encoding for enzymes of fructan synthesis, neither to insufficient sucrose levels to induce fructan synthesizing enzymes in stems. Evidence is presented indicating that the main fructan synthesizing enzymes (1-SST and 1-FFT) are indeed expressed in sunflower, and that 1-Kestose (i.e., the product of SST activity), is also detectable among sugars stored in the stem. However, fructan amount appears to be only marginal, and its accumulation is likely not to be induced by treatments promoting high sucrose concentrations in the stem. These findings suggest that sunflower might potentially constitute a fructan-bearing crop, which might have an important impact from an agronomic point of view. An increase in grain yield and oil content, a more convenient fatty acids composition and, specially, an improvement in yield stability against drought and other abiotic stresses, could hypothetically be achieved if substantial amount of fructans could be accumulated in the sunflower stem. However, a large effort is needed to elucidate how sunflower potential to accumulate fructans is effectively elicited. A close examination of the regulatory mechanisms involved in its synthesis may be a first step toward achieving this goal.

## Conflict of Interest Statement

The authors declare that the research was conducted in the absence of any commercial or financial relationships that could be construed as a potential conflict of interest.

## Footnotes

1 http://www.asagir.org.ar/Asagir2008/importancia-economica.asp

2 http://atgc-sur.inta.gob.ar/welcome/default/index
